# HIV Genetic Diversity and Drug Resistance

**DOI:** 10.3390/v2020503

**Published:** 2010-02-02

**Authors:** André F. Santos, Marcelo A. Soares

**Affiliations:** 1 Laboratório de Virologia Humana, Universidade Federal do Rio de Janeiro, CCS, Bloco A, sala A2-120, Cidade Universitária, Ilha do Fundão, 21949-570, Rio de Janeiro, RJ, Brazil; E-Mail: andre20@globo.com; 2 Programa de Genética, Instituto Nacional de Câncer, Rio de Janeiro, Brazil

**Keywords:** HIV-1, drug resistance, genetic diversity, subtypes, clinical response

## Abstract

Most of the current knowledge on antiretroviral (ARV) drug development and resistance is based on the study of subtype B of HIV-1, which only accounts for 10% of the worldwide HIV infections. Cumulative evidence has emerged that different HIV types, groups and subtypes harbor distinct biological properties, including the response and susceptibility to ARV. Recent laboratory and clinical data highlighting such disparities are summarized in this review. Variations in drug susceptibility, in the emergence and selection of specific drug resistance mutations, in viral replicative capacity and in the dynamics of resistance acquisition under ARV selective pressure are discussed. Clinical responses to ARV therapy and associated confounding factors are also analyzed in the context of infections by distinct HIV genetic variants.

## 1. Introduction

### 1.1. Genesis of HIV Types and Groups

The human immunodeficiency virus (HIV) has been characterized in 1983 as the causative agent of the acquired immunodeficiency syndrome (AIDS) [1,2], although its presence in the human population has been dated back to between the 1900s and early 1920s [3,4]. From 1981 to 2008, 58 million people were estimated to be infected with HIV, 25 million of which have died [5]. AIDS can be considered one of worst pandemics in history, only paralleled to the Spanish influenza pandemic, which killed 20–40 million people [6].

HIV belongs to the *Retroviridae* family, and to the Lentivirus genus. This genus comprises both types of HIV (HIV-1 and HIV-2), in addition to a large number of simian immunodeficiency viruses (SIV) that infect naturally different non-human primate species in the African continent [7,8]. In their natural host, SIV rarely causes the collapse of immune system, and the detailed characteristics of this pathogen-host relationships remain unclear. The origin of HIV in humans was traced to multiple zoonotic infections, likely due to contact of hunters and butchers with corporal fluids of non-human primates infected with SIV [9,10]. The two HIV types have distinct origins. HIV-1 strains are closer to SIV that naturally infect chimpanzees (SIVcpz) [11], while HIV-2 strains are related to SIV from sooty mangabeys (SIVsm) [12]. The precise number of times SIVs crossed the specie barrier and infected humans with success will never be known, since many factors can influence the success of establishing infection, including: (a) the efficiency of transmissibility; (b) the capacity of avoiding the immunologic system; (c) a successful replication in the new host and (d) a pathogenic potential to guarantee human to human passage. However, the emergence of new HIV types or groups through novel transfer events of an SIV strain to humans is an ongoing possibility. In Central Africa, a recent work identified SIV circulating in risk groups that frequently have contact with corpses of primates killed by hunting [9]. HIV types have distinct patterns of spread and progression to AIDS. HIV-2 infection, for example, is restricted to regions of Western and Central Africa and account for few cases outside that continent, representing only 1.4% of the HIV isolates characterized (Table 1).

**Figure 1.**
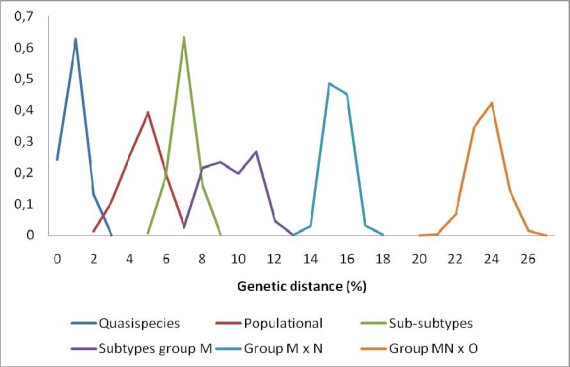
*pol* gene nucleotide distance within HIV groups, subtypes, sub-subtypes, populations and intrahost quasispecies. Estimates are presented as percentages of distances.

HIV-1 is responsible for the AIDS pandemic and can be divided into four groups (M, N, O and P), each one derived from a distinct introduction of SIVcpz in the human population (Table 1) [11,13]. HIV-1 group O (Outlier) is the most divergent group (Figure 1), and it has recently been suggested it could have originated from SIV that infected wild gorillas (SIVgor) [13]. SIVgor is related to SIVcpz and thus it is possible that gorillas are an intermediate reservoir of HIV-1 group O [14]. The HIV-1O epidemic pattern is restricted to the West and Central Africa, and around 15,000 people are estimated to be infected with this variant in Cameroon [15,16]. Group N (New) was only identified in 1998 [17], and its origin was traced to a recombination event between the ancestor of group M and SIVcpz [18]. HIV-1N is very rare epidemiologically and less than 50 cases have been identified to date, all in Cameroon [19,20]. Recently, a new HIV-1 group (P) was described in a Cameroonian woman [21]. The origin of this group was correlated with SIVgor, without evidence of recombination with other HIV-1 groups. Currently, this is the only identified case of this variant, but other cases are expected to be occult in Cameroon. HIV-1 group M (Major) alone is responsible by more than 95% of the AIDS pandemic and virtually all studies in HIV research have been conducted with representatives of this group.

### 1.2. HIV-1 Group M Diversity

In the early 1990s the sequencing and alignment of viral genes *env* and *gag* from different strains of HIV-1 allowed for first time to establish the presence of well-defined HIV-1M genetic clades. Based on this information, subtypes A, B, C, D, E and F were recognized in 1993 [22]. In 1994, subtypes G and H were characterized in Central Africa, and later subtypes I (1995), J (1999) and K (2000) were described [23–26]. In the *pol* gene, the most conserved in retroviruses, the nucleotide divergence between HIV-1 subtypes is around 9–11% (Figure 1).

With the advance and popularization of DNA sequencing techniques, new genomic regions were obtained and analyzed, including HIV-1 full-length genomes. All HIV strains previously classified as subtype E based on *env* gene phylogeny had divergent subtype A classification in *gag* and *pol* [22,27], revealing that HIV-1 can generate inter-subtype recombinant strains. Indeed, a “pure” subtype E has not been found to date. Other cases involving recombination were described for subtype G, in which some mosaic strains were classified as *env* A/*gag* G primarily in Central Africa [22,28,29]. The “pure” subtype I was evidenced as being a complex recombinant involving subtypes A, G and I [30,31].

According to the current classification, HIV-1 group M is divided into nine different “pure” subtypes or non-recombinant forms (A-D, F-H, J and K) [32]. Intersubtypic mosaic isolates are divided into two distinct categories: when it is found in a population (in at least three people with no epidemiologic link and with the same intersubtypic breakpoints), this form is classified as a circulating recombinant form (CRF); conversely, when it is found only in a single patient, it is classified as a unique recombinant form (URF). Currently, over 40 CRFs have been characterized. “Pure” subtypes E and I have been reclassified to CRF01_AE and CRF06_cpx, respectively. Some subtypes are further divided into sub-clusters denominated sub-subtypes. Genetic differences between sub-subtypes are around 7% in *pol* (Figure 1), and to date only two subtypes are divided in sub-subtypes, subtype A (A1 through A5) and F (F1 and F2) [33–35].

**Figure 2.**
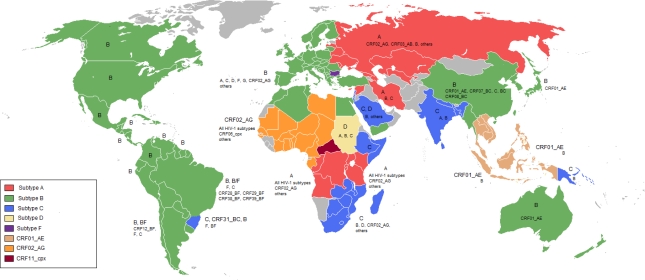
Worldwide prevalence of HIV-1 group M subtypes and CRF.

The worldwide distribution of HIV-1 subtypes is very heterogeneous, with regional prevalence of specific variants (Figure 2). The most prevalent HIV-1 form is subtype C, which in 2004 was responsible for about 50% of the global infections [36]. It prevails in sub-Saharan countries (where two-thirds of all infected people are located), in Eastern African countries, in the highly populated India and neighbor countries and in the southern region of Brazil. Subtype A is prevalent in Central Africa, Iran and Eastern Europe, and in Central Asia. In all these cases, sub-subtype A1 is more prevalent, whereas sub-subtypes A2 and A3 are primarily found in Africa and rarely in Europe. Subtype B is the most disseminated variant. It predominates in developed countries, such as the United States, countries of Western Europe, Japan and Australia. The remaining six HIV-1 subtypes (D, F, G, H, J and K) represented together only 10% of new infections in 2004 [36]. Some CRFs have great impact in local AIDS epidemics, such as CRF01_AE in Southeast Asia and CRF02_AG in Western Africa. CRF06_cpx is the second most prevalent recombinant form in West Africa, while others have negligible epidemic relevance.

HIV-1 molecular epidemiology is dynamic over time. For example, subtype C was dated to have entered Brazil in early 1980s [37–39], and recently this subtype was shown to represent 50% of new infections in the Brazilian southern region and 5%–30% in neighboring states [37,40–43]. While this subtype is spreading in Brazil, it overgrew other subtypes in Sub-Saharan Africa practically to extinction, such as subtype B and D, which were common in that region in the early 1980s [44,45].

### 1.3. Antiretroviral Treatment Against HIV and Drug Resistance

In March of 1987, AZT (zidovudine) was the first HIV-1 drug approved by the U.S. Food and Drug Administration (FDA) for AIDS treatment. Between 1991 and 2007, 24 additional drugs were approved, divided into five classes, according to the viral protein target (Table 2). Currently, the treatment indicated is a combination of drugs, named *highly active antiretroviral therapy* (HAART), which consists of at least three different drugs, two nucleoside reverse transcriptase inhibitors (NRTI) and a third, non-nucleoside reverse transcriptase inhibitor (NNRTI) or a protease inhibitor (PI). Due to high costs, more recent antiretroviral drug classes, such as entry inhibitors (EI) and integrase inhibitors (II), are primarily used for salvage therapy in cases of multiple resistances to the previous classes [46].

HIV eradication from infected subjects is still a distant scenario, but the currently available treatment provides better quality of life to HIV/AIDS patients, minimizing viral replication, promoting partial recovery of the immune system and, thus, delaying or halting disease progression [46]. On the other hand, there are many obstacles to therapeutic efficacy in patients, including several adverse effects, limitation of drug penetration in particular reservoirs of virus replication, individual pharmacokinetics, treatment adherence, co-infections with other pathogens and the emergence of drug resistant viral strains.

With the advent of HAART, effective viral load suppression can be maintained for many years [47,48]. This is a clear advance compared to the AZT monotherapy of the early days, when many patients presented drug resistance within a few months of continued therapy [49,50], or to dual NRTI therapy, when two years of treatment resulted in viral load rebound [51]. A key factor for the appearance of viral resistant strains is the retroviral reverse transcriptase (RT). It lacks an exonuclease proofreading system characteristic of cellular DNA polymerases, and is therefore prone to nucleotide misincorporation during cDNA synthesis, which persists in the newly synthesized copy and is integrated into the host genome, producing a new variant. This repair inability of HIV RT confers a high mutational rate to the virus (approximately 5–10 nucleotide misincorporations per genome per viral cycle) [52]. Another important characteristic of HIV is its large progeny, with the generation of around one billion new viral particles a day in an infected individual [53]. These two factors together promote high viral plasticity, leading to the presence of a viral quasispecies within patients, frequent escape from the host immune system, and the emergence of resistant strains under drug selective pressure [54].

Currently, drug resistance mutations (DRM) have been described for all drugs in clinical use. In the polymerase domain of RT 13 positions were correlated with loss of drug susceptibility to NRTI, and 14 positions to NNRTI. In the protease (PR) region, 38 positions interfere with PI susceptibility. Seven positions in gp41 are associated with fusion inhibitor resistance and three positions in the viral integrase are related to II [55]. Two types of drug resistance mutations are recognized. Those mutations whose presence alone causes great loss of susceptibility to one or more drugs are called major or principal mutations. In some cases, a single mutation can lead to cross resistance to all drugs within a given drug class. This is the case for L100I and Y181I/C mutations in RT, which confer resistance to all NNRTIs, and for I84V in protease that results in loss of activity of five out of eight available PIs [55]. Usually, these major DRMs are located close to the active site of the target viral enzyme. Enzymes with major resistance mutations are less fit than their wild-type counterparts [56–60]. The lost replicative capacity of the mutant enzyme is partially or fully recovered by the acquisition of compensatory or accessory DRM [56,61–63]. It is noteworthy that the acquisition of two or more accessory mutations sometimes also be implicated in the loss of drug susceptibility [55]. Of note, some mutations considered major to one or more drugs can act as accessory DRM to others, such as L90M, which is considered major to NFV and SQV and compensatory to all remaining PIs, with the exception of DRV [55].

## 2. The challenge of HIV-1 non-B subtypes

An important issue extensively discussed in the last years relies on the impact of ARV drugs in different HIV-1 subtypes. This concern is based on the fact that the great majority of the studies conducted on HIV drug design, acquisition of drug resistance mutations, genotyping for drug resistance evaluation, and the phenotypic impact of DRM on HAART have been performed primarily for subtype B strains. Such concern has been particularly strengthened by two reasons. The first is the introduction of HIV treatment in African countries, where more than two-thirds of the people with HIV/AIDS live and where the HIV epidemiology is largely dominated by non-B HIV-1 subtypes (Figure 2). The second reason is the spreading of non-B subtypes in areas formerly recognized as being predominated by subtype B. In some Western European countries, the proportion of HIV-1 non-B subtypes is greatly increased. In Portugal, for example, subtype B represented 42% of new infections in 2003; subtype G alone was responsible for 29% and other subtypes (C and F) and recombinants forms (CRF02_AG and mosaics) for another 29% of new infections [64]. In the United Kingdom, subtype B accounted for 52% of the infections, subtype C for 21%, and subtype A for 9%, while other subtypes (D, F, G, H and J) and recombinant forms were responsible for 18% of new infections [65]. Tatt *et al.* (2004) showed that subtype C was responsible for 35% of new infections in heterosexual patients in England and Wales in 2000, while subtype B represented only 25% and subtype A, 15% of the infections [65]. In France, 24% of new infections were attributed to non-B subtypes (especially CRF02_AG) in 2001–2002 [66]. In Greece, subtype A was responsible for 42% of new infections in 2004 *vs.* 33% for subtype B infections [67].

HIV-1 non-B subtypes represent a challenge to HAART treatment as there are scarce studies in the literature on the efficacy of treatment in the context of infection by these subtypes. Different HIV groups and subtypes carry in their genomes genetic signatures and polymorphisms that could alter the structure of viral proteins which are targeted by drugs, thus impairing ARV drug binding and efficacy. HIV-2, for example, is less susceptible to some PIs, such as amprenavir, ritonavir and indinavir [68,69]. AZT also appears to be less efficient in this HIV type [68,70]. All NNRTIs are equally innocuous against HIV-2 [71]. The same was observed for some HIV-1 group O isolates that presented naturally a cysteine at RT position 181 (181C), considered a major DRM to NNRTI [54]. The same study also showed the secondary NNRTI DRM 98G as a natural polymorphism in group O [72]. Indeed, such isolates have presented a high natural resistance to this class of inhibitors.

Various non-B HIV-1 group M subtypes present genetic signatures and polymorphisms in their protease which are considered as compensatory DRM in subtype B (Table 3). These differences have triggered discussions as to whether those non-B subtypes are naturally less susceptible to PIs, which could in turn compromise the use of PI-containing HAART regimens [73–77]. The acquisition of various compensatory DRM can lead to therapeutic failure to PIs [55] and, on their turn, non-B subtype infections could fail treatment faster than those with subtype B infection. It is important to emphasize that no major DRM is naturally found present in HIV-1 non-B subtypes, and these concepts apply exclusively to compensatory mutations.

## 3. Drug Susceptibility in Treatment-Naive Patients Infected with HIV-1 non-B Subtypes

In recent years, a few studies have been conducted on drug susceptibility of different HIV-1 subtypes from treatment-naïve subjects. They have shown that drugs belonging to the PI, NRTI and NNRTI classes are highly efficient in inhibiting HIV-1 non-B subtypes [78–84]. There was no evidence that the presence of signatures and polymorphisms in those subtypes cause natural resistance to antiretroviral drugs. However, it has been reported that different non-B subtypes, notably subtypes C, F, G and CRF02_AG, presented different susceptibility to specific PIs [82,83]. A study has shown that subtypes C and G were more susceptible to IDV than subtype B, while CRF02_AG was more susceptible to NFV and RTV than subtypes B, C, F and G. Polymorphisms at PR positions 19, 35, 37, 70 and 89 were associated with drug susceptibility and different subtypes through Bayesian network analysis [83]. As all PI were designed for subtype B it seems paradoxical that some subtypes are more susceptible than the original target. With respect to the newer PI tipranavir, subtype F appears to be twofold less susceptible than other subtypes [82,83], while all subtypes are highly susceptible to darunavir [82]. About 10% (4/42) of subtype G isolates presented a natural lack of susceptibility to at least one PI, in the absence of a recognized DRM [80,84]. No case of natural resistance to PI analyzing 31 subtype C treatment-naïve isolates [80,81], as well as 16 CRF02_AG drug-naïve isolates, has been found [80,84]. The remaining non-B subtypes have only a few drug-naïve isolates phenotyped, and no robust conclusions can be drawn. Additional studies are necessary to unequivocally elucidate the issue of natural resistance in HIV-1 non-B subtypes and the determinants affecting drug susceptibility in these variants.

## 4. Selection of Major Drug Resistance Mutations in HIV-1 Non-B Subtypes

With the advent and dissemination of HAART in countries where HIV-1 non-B subtypes are prevalent, studies in DRM acquisition have accumulated over recent years. Viral isolates of HIV-1 non-B subtypes from patients under therapeutic failure primarily contain the major DRMs known for subtype B to PR and RT inhibitors [85–90]. On the other hand, the proportion of definite DRMs can differ among subtypes. For example, it is common to find the major DRM D30N in subtype B PR under nelfinavir exposure, but this mutation is rarely found in other subtypes [81,89,91]. A recent work reported that subtype F1 appears to acquire a low proportion of M46I and I84V compared to subtype B [92]. Such reports are in agreement with results of a global collaboration in PR and RT genotypes [89], where some mutations were found at a low frequency in the PR of specific non-B subtypes: M46L (subtype C), I84V (subtypes C, F and G) and L90M (subtypes A and F). Conversely, V82T was more frequent in subtype G.

With respect to NRTIs, the frequency of mutations related to resistance to thymidine analogues (TAM) also show differences between subtypes. There are two well known distinct resistance pathways to thymidine analogues: TAM-1, which includes mutations M41L, L210W and T215Y; and TAM-2, characterized by mutations D67N, K70R, T215F and K219Q/E [55]. In some cases, a mixture of these mutational pathways can be observed. In subtype B, mutations were more frequently related to the TAM-1 than to the TAM-2 pathway [89], although mutations correlated with both TAM pathways were generally more frequent in subtype B than in other HIV-1 subtypes. Conversely, a study with subtype C viruses in AZT/ddI-containing HAART has shown the preferential acquisition of a mixed TAM pathway comprising mutations D67N, K70R and T215Y [93]. A recently published study in Burkina Faso showed a differential proportion of DRM in subjects infected with CRF02_AG and CRF06_cpx, the latter being the more prevalent form in that country [94]. Interestingly, CRF06_cpx appeared to accumulate NRTI DRM faster than CRF02_AG. Twenty five (69%) patients with CRF06_cpx presented three or more TAMs, while only three (10%) patients with CRF02_AG presented the same TAM numbers. Both forms presented the TAM-1 as the preferential TAM pathway.

Treatment regimens containing the NRTI 3TC, ABC, FTC, TDF and ddI can select the uncommon DRM K65R [55]. This mutation is more prevalent in subtype C than in subtype B, and it is more prevalent in both latter subtypes than in subtype A [95,96].

With respect to NNRTIs, a study among pregnant women using single-dose NVP for mother-to-child transmission prophylaxis in Uganda and Malawi showed that the proportion of viral resistance to that drug was higher in subtype C (69.2%) than in subtype A (19.4%) and D (36.1%) isolates [97]. The proportion of NVP-resistant viruses in the newborns was also higher in those infected with subtype C than with subtypes A and D [98]. Mutations K103N, Y181C and Y188C were more prevalent in subtype C than in subtypes A and D [97,99].

Some of the results described above remain inconclusive because there was no assessment of the extension of treatment exposure and the specific drug regimens of the individuals analyzed. Differences of B and non-B subtypes in acquiring specific DRM could be due to differences in those parameters; for example, more prolonged exposure of patients infected with subtype B to monotherapy and dual therapy, mostly available in subtype B-predominating countries in the first decade of the HIV/AIDS pandemic. As the use of HAART is more recent in developing countries, non-B subtypes have been less exposed to mono- and dual therapy, and may acquire DRM at a slower pace. At the same time, other factors can influence such differences, such as viral fitness in the presence of DRM, genetic barriers, kinetics of DRM emergence, subtype-specific mutations (SSM), polymorphisms and differential impact conferred by the same DRM in different subtypes. Those factors will be discussed in further detail below.

## 5. Drug Resistance Mutations and Fitness

Although many compensatory DRM can be naturally found in treatment-naïve isolates as polymorphisms or genetic signatures in different HIV-1 subtypes, the sites at which major DRM occur are highly conserved in HIV-1 group M isolates. The acquisition of viral resistance often implies structural modifications within or close to the enzyme’s active site, allowing the protein to better discriminate its natural substrate in the restrictive environment imposed by drugs. The cost to this advantage is generally paid through loss of replicative capacity. The frequency of major DRM can be inversely correlated with its cost to viral fitness, *i.e.*, the higher the cost, the lower the frequency. For example, in a Canadian study analyzing first line HAART regimens containing AZT, ddI and NVP, 86% of the patients presented resistance to NVP, while only 5% presented resistance to ddI and 19% to AZT after 30–60 weeks of treatment [100]. The genotyping of those isolates revealed that the most frequent mutation was K103N, followed by G190A and Y181C. A study conducted by Collins *et al.* showed that K103N-containing viruses were fitter than those containing Y181C, G190A or V106A [101]. With respect to PR resistance, a study conducted with patients under PI-based HAART showed that L90M was the commonest DRM (30%), independently of the PI used and of the number of PI-containing regimens, followed by V82A/F/T (20%) and D30N (16%). G48V and I84V were less frequently seen (<5%) [102]. In this sense, Mammano *et al.* showed that L90M does not severely affect viral fitness and that V82A reduced it in about 10% compared to wild-type viruses. On the other hand, the presence of G48V impaired viral replication by more than 50% [103]. Another study corroborated the reduced replicative loss of L90M-containing viruses and showed that the presence of D30N clearly affected replicative capacity [104]. It is worthwhile mentioning that all those experiments were conducted in HIV-1 subtype B. Therefore, the possibility that the fitness cost of the same mutations is different in other HIV-1 subtypes cannot be ruled out.

Evidence that different HIV-1 subtypes can present distinct resistance fitness in the presence of drugs was observed, for example, for NFV. According to the IAS consensus, viral isolates under NFV pressure can select D30N or L90M [55]. A study by our group showed that subtype B viruses with D30N or L90M had only marginal loss of replicative capacity (RC), of about 10% in relation to the wild type virus. On the other hand, the impact was more severe in subtype C, in which the presence of L90M caused a loss in RC of 20%. Subtype C with D30N alone was not able to replicate in cell culture [105]. The high replicative cost of D30N in subtype C could be correlated to its low acquisition in that subtype.

Another well documented example of how specific mutations can impact differently on virus replication is that caused by TAM pathways in HIV-1 subtypes B and C. Differences were described for the impact on fitness when a single TAM was introduced in these subtypes [106]. The presence of mutations D67N and K70R did not affect the fitness of subtype B, but increased the replicative capacity in subtype C. Similarly, the double mutant M41L/T215Y did not affect subtype C, but it impacted on the fitness of subtype B. When triple mutants were compared, the results were also distinct. In subtype B, the fitness ranking order was: WT > TAM-1 (M41L, L210W and T215Y) > TAM-2 (D67N, K70R and T215F) = TAM-1/2 (D67N, K70R and T215Y). This evidence may correlate to the fact that TAM-1 is more frequently seen in subtype B isolates (see previous section). However, in subtype C, the ranking order was: TAM-1 > WT > TAM-1/2 > TAM-2. In this case, the explanation for the higher frequency of mixed TAM in subtype C [93] was attributed to fact of D67N and K70R, both TAM-2 mutations that confer higher replicative capacity to this subtype, are selected earlier than TAM-1 mutations. The fact that the mixed TAM is fitter than TAM-2 explains its higher prevalence in subtype C.

It is conceivable that the fitness cost is a potential cause of some differences in DRM acquisition in distinct HIV-1 subtypes. However, studies in this theme are still scarce in the literature. Future studies with new infectious clones of different HIV-1 subtypes will help to elucidate this interesting subject.

## 6. Genetic Barriers in Different HIV-1 Subtypes

The genesis of drug-resistant variants is caused by random mutation events in viral genomes during the reverse transcription step of the virus life cycle. The change of a nucleotide at a definite codon can translate into amino acid changes. Therefore, the frequency of a DRM could be directly related to the number of modifications in the genetic code needed for the acquisition of the amino acid conferring resistance. HIV-1 subtypes have silent mutations in their coding sequence that could favor the emergence of distinct mutations in a definite subtype or group of subtypes (Table 4). In the PR region, the frequency of a single mutation can be explained by genetic barrier. In subtypes B, C and F, the resistant variant of codon position 82 is an alanine (A), while in subtype G it is a threonine (T) [89]. Treatment–naïve isolates of subtype G usually have an isoleucine (I) at PR position 82, while all other subtypes carry a valine (V). The change of 82I into the resistant form 82A needs two transitions, while it requires only one transition to a change into 82T. On the other hand, it is easier for other subtypes to change its natural form 82V to 82A (one transition) than to 82T (two transitions) [107]. In the RT region, other DRM have been correlated to specific subtypes and genetic barriers, such as the case of mutations Q151M and L210W and their low frequency in subtype F [89,107–109]. In addition, this was also observed for L210W in some isolates of subtype C (30%) and more frequently in subtype G and CRF02_AG (above 80% of the isolates) [89,107]. A yet another example is described for V106M, a cross-class NNRTI resistance mutation rarely found in clinical studies in most HIV-1 subtypes, with the exception of subtype C [89,110–113]. In this case, subtype C needs only a transition to acquire V106M, while all remaining subtypes require two transitions [106,110].

Yet another interesting case of genetic barrier for DRM and subtypes is the NRTI mutation K65R. It was shown that in HIV-1-infected cell cultures treated with TDF, that mutation was selected more rapidly in subtype C (12 weeks) than in subtype B (34–78 weeks) [114]. In the same experiment, viruses of subtypes A, G, CRF01_AE and CRF02_AG did not develop K65R during 30–33 weeks. The impairment caused by this mutation in the replicative capacity was similar in subtypes B and C. All the viral isolates tested in this study were derived from samples isolated from patients’ peripheral blood mononuclear cell (PBMC) co-culture, and therefore were clinically relevant. Another study by the same group showed faster acquisition of K65R in subtype C in cell culture with ABC (22–30 weeks), ddI (22–30 weeks) and TDF (12 weeks), while subtype B and HIV-2 isolates did not select for this mutation after 22–30 weeks in the presence of these drugs [115]. The combination of two NRTIs in the same cell culture also resulted in K65R acquisition in subtype C isolates in 12–30 weeks, but not in subtype B or HIV-2 isolates. Both subtypes B and C need only a transition to acquire this mutation. In this case, the explanation relied on differences at polymorphic sequences at RT codons 64 and 65 in these subtypes. Subtype B has AGG AAA at these codons, while subtype C has AAA AAG. This adenine-rich run exclusively present in subtype C appears to cause a pause in RT during the (+) strand DNA synthesis step, favoring nucleotide misincorporation and increasing the chance of K65R occurrence [116]. Clinical studies corroborated these data, showing a higher accumulation of K65R in subtype C [117–119]. This is the first example of the impact of silent mutations outside a resistance codon which are correlated with the acquisition of drug resistance in HIV.

Genetic barrier may explain some cases of differential emergence of DRM. Nonetheless, the mutational cost to acquire major DRM is generally similar in the most prevalent HIV-1 subtypes and CRF (A-D, F, G, J, CRF01_AE and CRF02_AG). This indicates that genetic barrier is not the sole cause of differences in DRM acquisition observed among HIV-1 subtypes.

## 7. Kinetics of DRM Emergence

Another potential mechanism to explain distinct prevalence of DRM between subtypes is differential kinetics for DRM acquisition across drug exposure time. However, there are only a few studies on this issue to date. Such studies nevertheless showed some interesting differences. For example, in Brazil, it has been shown that subtype C isolates from patients under antiretroviral treatment accumulate lower rates of DRM to NRTIs and PIs than isolates of subtype B [120]. After five years under NRTI-containing HAART, 45% of subtype B isolates presented at least one major DRM to that class, while subtype C isolates presented only 19% in the same period. For PI, after the same time, 36% of the subtype B isolates had at least one major DRM *vs.* only 6% of subtype C viruses. The kinetics of DRM acquisition was similar in both subtypes for NNRTIs. Another study showed that isolates of sub-subtype F1 under NFV-containing regimen accumulates L90M slower than subtype B (5% *vs.* 16%, respectively) after three years of treatment [92]. However, subjects infected with subtypes B and G from Portugal did not present differences in the proportion of resistant strains after four years under a NFV-based HAART regimen [121]. In this case the lower presence of D30N in subtype G was compensated by higher acquisition of L90M. On the other hand, subtype G had a lower frequency of IDV resistance (20%) than subtype B (40%) after six years of treatment.

The explanation behind this phenomenon remains unclear. Studies about fitness in major DRM for non-B subtypes are yet scarce as previously discussed. These studies show that in some cases, non-B subtypes had a lower frequency of DRM acquisition across treatment exposure time than subtype B, for which all available drugs have been designed.

## 8. HIV Subtype-specific Mutations

Another possible explanation for the differential kinetics of DRM acquisition between subtypes would be the emergence of subtype-specific mutations (SSM) that compensate for the lower proportion of recognized DRM compared to subtype B. The first SSM described was M89I/V for subtypes C, F and G [122]. This mutation was more frequent in patients failing PI-based HAART with major DRMs. However, phenotyping of isolates carrying M89I/V characterize it as a secondary DRM. Isolates of subtypes C, F and G with M89I/V and L90M had two-fold decreased susceptibility to NFV compared to that of isolates carrying L90M alone. Bayesian analysis correlated these mutations with A71T and T74S. Another work showed that PR clones of subtype G isolates without these latter mutations reversed 89I/V to 89M [123]. More recently, we showed that M89I is essential in subtype G to confer high level of resistance to NFV and SQV in clinical isolates with L90M [122].

Other mutations have also been correlated with treatment, such as those at PR positions 6 (subtype C), 15 (CRF02_AG), 19 (subtype F), 37 (subtype A) and 64 (subtype C and CRF01_AE), in addition to RT position 102 in subtype C [89]. However, their actual role in drug resistance remains unknown. Another recent study has associated the PR substitutions V13A (IDV, APV and LPV), D35N (NFV), I66F (IDV) and I82M (IDV and LPV) with PI treatment in subtype G by regression analysis [124].

Subtype-specific mutations require careful phenotypic characterization, since the identification of mutation as a major DRM will drastically change the resistance genotyping interpretation for a given subtype or group of subtypes. Most importantly, the correct interpretation of SSM will better guide treatment in countries where non-B subtypes prevail.

## 9. Polymorphisms and Signatures in Non-B Subtypes Correlated with DRM

HIV-1 subtypes have genetic signatures and amino acid polymorphisms that cause conformational changes at their proteins and such hindrances can facilitate or impair the acquisition of definite DRM. Interesting examples are mutations N88D and N88S in PR. They are both selected under NFV pressure. However, in clinical isolates of subtype B with at least either mutation D30N, N88any or L90M, or a combination of these, N88D was present in 21.2% of patients, compared to a prevalence of N88S in only 5.2% of patients [125]. N88D is classified as compensatory DRM and is strongly correlated with the D30N resistance pathway. Subtype B isolates with D30N and N88D are fourfold less susceptible to NFV (mean fold-change (FC) = 50) than isolates with D30N alone (mean FC = 14). However, the presence of N88S was negatively correlated with D30N and isolates carrying N88S presented a mean FC of 11, similar to D30N, defining the former as a major DRM. N88S confers hypersusceptibility to APV [125,126]. In other clinical studies, N88S was more frequently found in isolates of CRF01_AE than in subtype B [89,91]. N88S was also more frequent in subtype F than subtype B in Brazil [92]. Molecular dynamics simulations with PR-NFV complexes of subtypes B (wt), B-D30N, B-N88S, CRF01_AE (wt), CRF01_AE-D30N and CRF01_AE-N88S showed discordant results [127]. In subtype B, D30N reduces the energy of binding to NFV more than does N88S. The opposite was observed in CRF01_AE, which could explain the differential prevalence of those mutations in these two subtypes. Proteases of CRF01_AE used in this study carried some natural polymorphisms (K20R, E35D and I93L) and signatures (M36I, R41K, H69K and L89M) that interfere in the differential acquisition of D30N and N88S, particularly the signature M36I [128]. This study hypothesized that such signatures make D30N less effective against NFV in non-B subtypes.

## 10. Differential Resistance Conferred by the Same DRM

The classification of DRM into major or compensatory mutations is exclusively based on data generated with subtype B. For example, mutation L90M in PR of subtype B is considered major to NFV and SQV and compensatory to other PI [55]. Other instances include most of the major DRM in PR: V32I, L33F, M46I/L, I47V, I50V, V82A/F/T/S and I84V. However, it is conceivable that such classification might not hold true for non-B subtypes. Because of subtype-specific amino acids and therefore structural backbones, the same mutation can confer different levels of susceptibility to a given drug. In isolates of subtype G under NFV-containing treatment the commonest pattern found was an association between M89I and L90M (21%), while L90M alone was seen in only 10% of patients [121]. Changes at PR codon 89 in subtype B isolates were not observed. L90M alone in isolates of subtype G conferred a mean twofold drop in susceptibility to NFV (FC = 2.1), while the same mutation in subtype B conferred a sevenfold decrease (FC = 7.6). However, subtype G isolates carrying M89I/L90M have an FC to NFV similar to that of L90M alone in subtype B. Interestingly, subtype G isolates with L90M alone or M89I/L90M did not show resistance to SQV, while in subtype B L90M alone was able to confer decreased susceptibility of about 3.5 times to that PI. Another mutation observed in this resistance pathway was I54V/L. Its presence with L90M doubled the FC to SQV, while I54V/L per se did not have any impact for that drug.

Differential resistant phenotypes have also been observed in subtypes B and C carrying the PR mutation T74S. This mutation was described as compensatory, increasing the replicative capacity of PI multiply-resistant viruses of both subtypes [129]. Its presence also increased the susceptibility to most PIs in subtypes B and C. However, some phenotypic effects diverged between them. Subtype B clones with DRMs M46I, I54V, V82A and L90M and with T74S showed a resensitization of the virus to IDV, while subtype C clones with the same mutations remained resistant to that drug. T74S in multi-resistant viruses also decreased the FC of subtype B to SQV (11.7 to 2.0), to LPV (12.8 to 2.8) and to RTV (240 to 7.0). However, such resensitization was less pronounced in subtype C to LPV (189 to 29) and to RTV (303 to 25.7).

In the viral RT, mutation K65R conferred a more pronounced resistant phenotype to ABC, 3TC and ddI in CRF01_AE than in subtypes B and C [114]. A recent report also showed differences in the resistance levels conferred by TAMs to AZT in subtype B and CRF01_AE [130]. The TAM-1 pathway (M41L, L210W and T215Y) conferred a higher fold-resistance to CRF01_AE (51×) than to subtype B (17×) compared to their respective wild-type clones. Similar results were obtained for the TAM-2 pathway (D67N, K70R, T215F and K219Q) in CRF01_AE (64×) and subtype B (13×). The cause of this difference was mapped to the change A400T in RT, a signature present in CRF01_AE. When the threonine was reverted to alanine in CRF01_AE RT, the resistance level was similar to that of subtype B. This represents the first experimental evidence that a natural signature directly influences resistance levels to antiretroviral drugs in different subtypes.

Subtype-specific protein backbones, in addition to affecting fitness or facilitating DRM acquisition, can also confer different resistance levels to definite HIV-1 group M subtypes. Studies in this area are novel and can help improve current genotyping algorithms, essential to guide better salvage regimens in patients failing therapy.

## 11. Impact of HIV-1 Subtype on Therapeutic and Clinical Response

With the expansion of antiretroviral treatment throughout the world, HAART has been introduced in countries where non-B subtypes are prevalent. This scenario created an opportunity to evaluate patients’ response with respect to HIV viral load (VL) decrease and CD4^+^ T-cell recovery in the context of distinct HIV-1 subtypes. A study in Europe compared treatment response of European patients, all infected with subtype B, with that of African immigrants, the majority of which infected with subtypes A, C and D [130]. For this comparison only patients in their first-line HAART regimen were enrolled. The time to HIV VL undetectability (<500 RNA copies/mL) was strikingly similar for both groups after 12 months of therapy (>90%). The CD4^+^ T-cell recovery was also similar between groups during the first 21 months of treatment. Another study in France with patients infected with subtype B (76%) and non-B (24%) in first-line regimen also failed to denote differences in HIV VL control and in CD4^+^ T-cell recovery [132]. In this study, however, “non-B subtypes” were pooled together, preventing the observation of any differences between the subtypes present. Other studies in developed countries corroborated the lack of influence of subtype on patient’s response to HAART [133–135]. A study in Angola with patients in first-line triple drug combination showed that a great part of them was under HIV VL control (74%) after one year of treatment, irrespective of the infecting subtype [136]. Interestingly, a recent work in England with patients infected with subtypes A, B and C showed that VL suppression occurred more rapidly in subtypes A- and C- than in subtype B-infected subjects [137]. Moreover, the latter showed higher CD4^+^ T-cell recovery rates compared to the remaining subtypes.

An important confounding factor present in several of the studies that compared the effect of subtype on treatment outcome is the ethnicity of the subjects. Even in cases where patients were enrolled from a single care site, differences in cultural aspects of individuals from diverse geographic locales or continents may play an important role in the outcome. Adherence to treatment is a clear example of those aspects. Indeed, one of the studies that found a “subtype effect” on treatment outcome was able to associate ethnicity with virologic responses [138], therefore preventing a clear distinction between the ethnicity and subtype factors.

HIV strains with higher genetic distances to HIV-1 group M seem to be more prone to failing clinical outcomes. A meta-analysis conducted with Belgian and Luxembourgian patients comparing clinical outcomes after 12 months of treatment upon HIV-1 or HIV-2 infection showed reduced immunologic and virologic responses in the latter group [139]. The use of PI improved the response of the HIV-2-infected patients as opposed to the exclusive use of RTI. This is an important drug combination against HIV-2, where NNRTIs are ineffective as previously discussed.

It is apparent from the abovementioned studies that first-line HAART regimens are similarly efficient in suppressing HIV VL and CD4^+^ T-cell recovery in patients infected with different HIV strains. Whether people infected with diverse subtypes have differences in clinical outcomes after long-term treatment remains to be elucidated.

## 12. Conclusions

As the technology for the achievement of HIV eradication and of the formulation of an effective sterilizing HIV vaccine are still beyond our knowledge, antiretroviral therapy is yet to be further disseminated in the next future, particularly in developing countries. As a consequence, the understanding of the impact of HIV genetic diversity on the development of drug resistance and on treatment outcomes is of pivotal importance. Such knowledge concerns not only to developing, but also to developed nations, where the proportion of new HIV infections by non-B subtypes is currently rising sharply. The use of antiretroviral drugs, originally developed for subtype B, is so far similarly promising in geographical areas where non-B subtypes circulate. However, only long-term studies assessing the susceptibility to antiretroviral drug regimens in settings where non-B subtypes occur will be of paramount relevance to trace the role of the described polymorphisms / molecular signatures in the dynamics of drug resistance acquisition. Such studies should resolve and unequivocally discard confounding issues such as ethnicity and adherence. They should also avoid pooling non-B subtypes as a unique genetic entity, as they are as distinct from each other as they are from subtype B. Initiatives to extend ARV access in the developing world should permit additional, more structured trials to evaluate these issues. Most importantly, the enormous reductions seen in mortality and morbidity of HIV-infected individuals in resource-poor settings consequent to ARV access constitutes the proof-of-principle that HIV genetic variation should not be a reason to withhold worldwide dissemination of treatment.

## Figures and Tables

**Table 1.** Summary of HIV types and groups.

**Type**	**Group**	**Origin**	**Isolates (%)^1^**	**Epidemiology**	**Comments**
HIV-1	M	SIVcpz	259,678 (98.2%)	All continents with exception of Antarctica	Major group responsible for the AIDS pandemic; more fit than HIV-1 group O and HIV-2.
	O	SIVgor or SIVcpz	1,095 (0.4%)	Majorly found in Central and West Africa	Naturally resistant to NNRTI; less fit than group HIV-1 M and HIV-2.
	N	Recombinant group M ancestor / SIVcpz	22 (<0.001%)	Only found in Cameroon	Very rare epidemically; few studies on drug resistance published.
	P	SIVgor	Single case	Undetermined	Described in 2009 in a Cameroonian woman. The actual number of infections is unknown.
HIV-2	—	SIVsm	3,593 (1.4%)	Mainly found in Western and Central Africa; some cases in Western Europe, India, United States, Brazil and Japan	Apparently slower progression to AIDS; less susceptible to some anti-HIV-1 drugs; naturally resistant to NNRTI.

1 Isolates sequenced and available at the Los Alamos HIV Sequence Database as of 18 July 2009.

**Table 2.** Summary of antiretroviral drug classes.

**Drug Class**	**Activity**	**Drugs**	**Release year**
Nucleoside/Nucleotide Reverse Transcriptase Inhibitors (NRTI)	NRTIs are mimetics of nucleosides/nucleotides and bind to the active site of the polymerase domain in the viral RT enzyme, inhibiting the synthesis of double-stranded viral cDNA	Zidovudine (AZT)	1987
		Didanosine (ddI)	1991
		Zalcitabine (ddC)	1992
		Stavudine (d4T)	1994
		Lamivudine (3TC)	1995
		Abacavir (ABC)	1998
		Tenofovir (TDF)	2001
		Emtricitabine (FTC)	2003
Protease Inhibitors (PI)	PIs are mimetics of viral peptides and bind to the active site of the protease enzyme, preventing viral maturation in a late step of virus life cycle	Saquinavir (SQV)	1995
		Ritonavir (RTV)	1996
		Indinavir (IDV)	1996
		Nelfinavir (NFV)	1997
		Amprenavir (APV)	1999
		Lopinavir (LPV/r)	2000
		Atazanavir (ATV)	2003
		Fosamprenavir (fAPV)	2003
		Tipranavir (TPV)	2005
		Darunavir (DRV)	2006
Non-Nucleoside Reverse Transcriptase Inhibitors (NNRTI)	NNRTIs are designed to bind to an RT hydrophobic pocket, modifying its structure allosterically and impairing the polymerase domain catalytic site	Nevirapine (NVP)	1996
		Delavirdine (DLV)	1997
		Efavirenz (EFV)	1998
		Etravirine (ETR)	2008
Entry Inhibitors (EI)	EIs are small peptides that bind to envelope viral proteins (gp41 or gp120) and prevent the fusion between viral envelope and cellular membranes or virus attachment to co-receptors in early steps of the virus life cycle	Enfurvirtide (T-20)	2003
		Maraviroc (MVC)	2007
Integrase Inhibitors (II)	IIs bind to the viral integrase and prevent the integration of the viral double-stranded cDNA into the host cellular genome in the early steps of the virus life	Raltegravir (RAL)	2007

**Table 3.** Genetic signatures and/or polymorphisms of HIV-1 non-B subtypes associated with resistance to PIs.

**Drug-associated mutation**	**Drugs**	**% in Subtype B**	**Signatures**	**Polymorphisms**
I13V	TPV	13%	90%–98% in subtypes A, G and CRF02	4%–78% in other subtypes non-B
K20I	ATV	2%	93%–98% in subtypes G and CRF02	1%–3.5% in subtypes A, F and CRF01
M36I	ATV, IDV, NFV and TPV	13%	81%–99% in several non-B subtypes	—
H69K	TPV	2%	96%–97% in subtypes A, C and G, CRF01 and CRF02	2% in subtype F
V82I	ATV	2%	87% in subtype G	1%–6% in several non-B subtypes
I93L	ATV	33%	94% in subtype C	5%–40% in several non-B subtypes

**Table 4.** Drug resistance mutation and genetic barriers for HIV-1 group M subtypes

**Codon Position (*pol* genomic region)**	**Resistance mutation**	**Codon change**	**Genetic barrier in non-B subtypes**
82 (PR)	V82A	GTC → GCC (1 ts)	G: 14% GTC
	V82T	GTC → ACC (2 ts)	Other subtypes: 87%–100% GTC
82 (PR)	I82A	ATC → GCC (2 ts)	G: 84% ATC
	I82T	ATC → ACC (1 ts)	Other subtypes: 0%–9% ATC
118* (RT)	V118I	GTT / GTC / GTA → ATT / ATC / ATA (1 ts)	G: 63% ATA
		GTG → ATA (2 ts)	Other subtypes: 0%–6% ATA
151 (RT)	Q151M	CAG → ATG (2 tv)	D: 37% CAA
		CAA → ATG (1 ts, 2 tv)	F: 83% CAA
			Other subtypes: 2%–12% CAA
210 (RT)	L210W		C: 30% TTA / CTG
		TTG → TGG (1 tv)	F: 50% TTA / CTG and 23% CTA
		TTA / CTG → TGG (1ts, 1 tv)	G: 82% TTA / CTG
		CTA → TGG (2 ts, 1 tv)	CRF02_AG: 83% TTA / CTG
			Other subtypes: 82%–87% TTG
106 (RT)	V106M	GTG → ATG (1 ts)	C: 83% GTG
		GTA → ATG (2 ts)	Other subtypes: 0–7% GTG
108* (RT)	V108I	GTA → ATA (1 ts)	G: 62% GTG
		GTG → ATA (2 ts)	Other subtypes: 88%–100%

Data extracted from reference [103];

* Secondary DRM; ts – transition; tv - transversion
